# Acute Pulmonary Edema During a Cesarean Delivery After an Adverse Drug Event

**DOI:** 10.7759/cureus.32876

**Published:** 2022-12-23

**Authors:** Laura Gonçalves, Mariana Luís

**Affiliations:** 1 Department of Anaesthesiology, Centro Hospitalar Universitário do Porto, Porto, PRT; 2 Department of Anaesthesiology, Hospital Central do Funchal, Funchal, PRT

**Keywords:** acute pulmonary edema, flash pulmonary edema, cesarean delivery, phenylephrine, adverse drug events, pregnancy

## Abstract

Acute pulmonary edema (APEd) is rare in pregnancy and in the postpartum period. An intermediate type of APEd characterized as a transudate with a protein concentration between that of cardiogenic and noncardiogenic APEd has been described in the literature. This transudate might actually be the result of capillary pressure having increased to a point of high-permeability edema and/or alveolar hemorrhage. Clinically, the presentation would be a dramatic form of APEd - flash pulmonary edema - characterized by a rapid accumulation of fluid within the lung's interstitial and alveolar spaces as a result of suddenly elevated cardiac filling pressures. Here, we present a case of a healthy pregnant woman who underwent cesarean delivery and developed a constellation of signs and symptoms, suggestive of an APEd, after a supratherapeutic bolus of phenylephrine. During the diagnostic excursion, bilateral parenchymal infiltrations suggestive of hemorrhage were observed on a computed tomography scan. This case highlights the high morbidity associated with adverse drug events and the imperative to prevent them. It also underscores the critical need for careful management of volume shifts and hemodynamics in full-term pregnancies.

## Introduction

Acute pulmonary edema (APEd) is a relatively uncommon cause of respiratory failure in pregnancy and in the postpartum period, with a reported incidence of 0.08% to 1,5% in a review of 62,917 pregnancies [1]. However, it represents a source of significant morbidity and mortality in obstetric and neonatal populations [2].

APEd has been traditionally classified as cardiogenic or noncardiogenic, with the difference based on the protein concentration of the transudate [3-5]. However, an intermediate type of pulmonary edema has been described, which has been hypothesized to be the result of a two-stage process [6]. First, pulmonary capillary pressure (PCP) gradually increases, creating a situation that corresponds to a low-permeability/cardiogenic form of APEd. This stage is followed by a high-permeability stage.

In experimental studies, a severely acute elevation in PCP can lead to increased permeability of the capillary wall and ultimately to blood-gas barrier stress failure [5]. This phenomenon is manifested by edema, hemorrhage, or both, and it may occur in patients with flash pulmonary edema [6]. This dramatic presentation of APEd is characterized by a rapid accumulation of fluid within the lung's interstitial and alveolar spaces, as a result of suddenly elevated cardiac filling pressures [7].

We present and discuss the case of a pregnant woman undergoing cesarean delivery who developed an APEd, with bilateral hemorrhagic parenchymal infiltrations, after a supratherapeutic bolus of phenylephrine.

## Case presentation

A healthy 26-year-old pregnant woman (39 weeks and 3 days) underwent an elective cesarean delivery due to breech presentation. A subarachnoid block (8 mg of bupivacaine 0.5% and 2.5 mcg of sufentanil; height 162 cm) was administered, and after 5 min, hypotension, nausea, and headache ensued. Ten milligrams of ephedrine were given without response. Subsequently, 2 mg of phenylephrine was incorrectly administered due to a dilution error that went undetected at the time. Hypotension continued to worsen and bradycardia developed, leading to 0.5 mg of atropine being administered. Soon after, the patient developed a dry cough and reported a headache. At this point, hypertension and sinus tachycardia were present and oxygen saturation dropped (minimum SatO2 92%). Crackles were present on auscultation, and hypoxemia was noted on analysis of blood gases (partial pressure of carbon dioxide (PCO2), pH, and other parameters were normal). No other signs or symptoms were found on clinical assessment. Supportive treatment was immediately initiated. It included oxygen by a non-rebreather face mask and intravenous furosemide at a dose of 40 mg, which allowed completion of the surgery. Meanwhile, the anesthesia team members (ATMs), an anesthesia nurse, and an anesthesiologist reviewed recent events and detected the dilution error. At our institution, drugs are generally prepared by the anesthesia nurse, and since phenylephrine is only available as a 10 mg/mL ampoule, this drug must be diluted twice. The first dilution yields a 1 mg/mL solution, and a second dilution yields a 0.1 mg/mL solution, which is normally used for boluses. For cesarian deliveries under spinal anesthesia, this preparation is done beforehand. However, in this case, only the first dilution was completed. In other words, the syringe containing 1 mg/mL of phenylephrine was used, thus, the delivered dose was 10 times higher than intended. As the ATMs were trying to correct the hypotension as quickly as possible, the drug was given without further checking.

**Figure 1:**
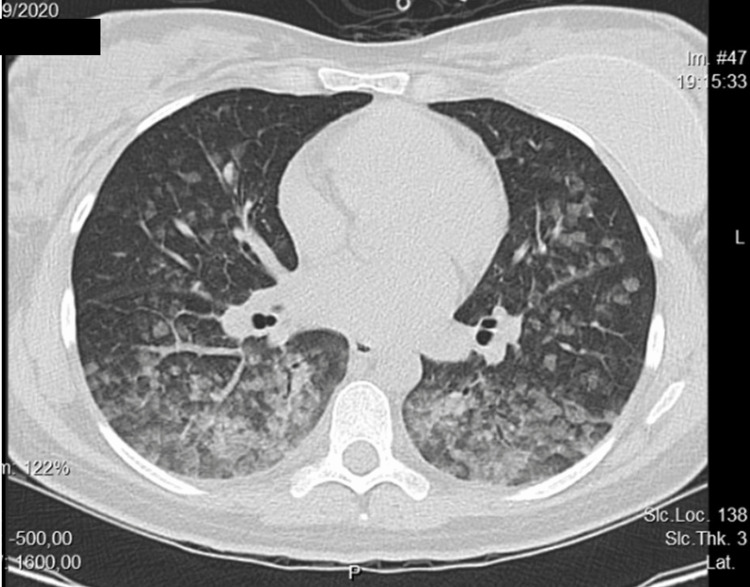
Thoracic CT scan, in a sagittal plane Bilateral pulmonary parenchymal infiltrations in a ground-glass pattern, more expressive on the upper segments of the inferior lobes, suggest bilateral hemorrhagic infiltration. A thin layer of bilateral pleural effusion is also seen.

This inadvertently higher dose of phenylephrine was thought to explain the constellation of signs and symptoms that were observed. Nonetheless, it was mandatory to exclude potentially life-threatening conditions, including APEd, myocardial infarction, and pulmonary embolism. The 12-lead EKG had no abnormalities. The blood analysis revealed normocytic, normochromic anemia and mildly elevated cardiac necrosis markers while N-terminal pro-brain natriuretic peptide (NT-Pro-BNP) was negative. Point-of-care cardiac ultrasound revealed hypokinesia of the inferior septal wall of the left ventricle, with no other changes. A computed tomography (CT) angiography revealed no sign of embolism. However, the CT demonstrated bilateral parenchymal infiltration in a ground-glass pattern, compatible with hemorrhage, as well as a thin layer of pulmonary effusion bilaterally (Figure 1).

Owing to these findings, the intensive care, obstetrics, and radiology teams were involved. The decision was made to have the patient remain in the postanesthetic care unit to receive monitoring and supportive treatment. A debriefing was performed by the ATM, under nonjudgmental terms, to address the dilution error and to delineate strategies to avoid its recurrence. Such strategies included label checks by a second person, implementing the “five rights” of administration (the right patient, the right drug, the right dose, the right route, and the right time), closed-loop communication while administering drugs, and discarding of drugs with indefinite content.

The patient’s condition evolved favorably within 24 h, in terms of clinical assessment and laboratory parameters. A CT scan was repeated on Day 4 and revealed the resolution of the described infiltrates. Blood markers for pulmonary capillaritis were all negative, including testing for antinuclear antibodies (extractable nuclear antigen screen, anti-double-stranded DNA, and anti-glomerular basement membrane antibodies) and perinuclear anti-neutrophil cytoplasmic antibodies (anti-myeloperoxidase and anti-proteinase 3). Discharge occurred six days after the event. An internal medicine appointment was set for a month later to follow up on the patient, but she missed it. To our knowledge, she did not require any other medical care, and the well-being of the neonate was not affected by this event. 

## Discussion

**Figure 2:**
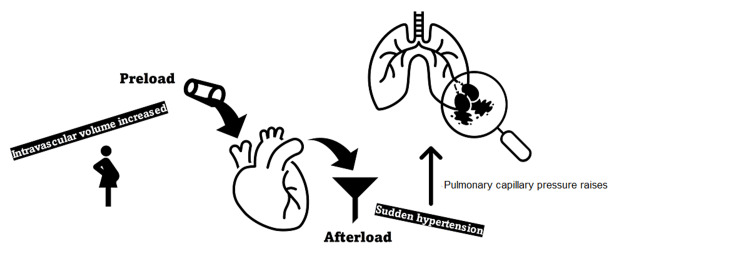
Schematic representation of physiological changes during pregnancy and predisposition to pulmonary edema During pregnancy, intravascular volume is increased (preload). If an additional factor, such as sudden hypertension (afterload) develops, this may result in pulmonary capillary pressure being increased to a point that leads to alveolar hemorrhage.

The physiologic changes associated with pregnancy, including an increase in intravascular volume (preload), may create a predisposition to APEd (Figure 2). Specifically, a hypertensive crisis (afterload) may cause a severe increase in PCP, resulting in alveolar hemorrhage [1,6]. Flash pulmonary edema is associated with a sudden rise in left-sided intracardiac filling pressures in settings such as a hypertensive emergency [7]. We hypothesize that a supratherapeutic bolus of phenylephrine, similar to the one described here, could be the precipitating factor for a blood-gas barrier stress failure.

Microscopic changes similar to a blood-gas barrier stress failure have been reported in acute pulmonary hypertension in animal models [6-8]. Unexpectedly, approximately 70% of such disruptions close within a few minutes of capillary transmural pressure reduction [9]. This rapid reversibility is consistent with our patient’s clinical evolution.

Presumably, the explanation for bleeding, rather than high-permeability edema, occurring after an increase in PCP lies in the very abrupt rise in PCP [8]. Alveolar hemorrhage syndrome emerged as a diagnostic possibility in our patient’s case, although bronchoalveolar lavage or lung biopsy was not performed. However, the three causes of alveolar hemorrhage syndrome are pulmonary capillaritis (discarded by serologic analysis), diffuse alveolar damage (characteristic of acute respiratory distress syndrome, which was unlikely), and bland pulmonary hemorrhage (causes include elevated left ventricular end-diastolic pressure, which was more likely).

Other possible diagnoses were also explored. Myocardial infarction was improbable since biomarkers were virtually negative and the 12-lead EKG was normal. Pulmonary embolism was excluded by a negative CT angiography. Takotsubo syndrome was also a possible diagnosis, but our findings did not meet all the criteria necessary to diagnose it [10].

There are reports described in the literature on pulmonary edema and other cardiac complications, even after topical administration of phenylephrine (ocular and nasal routes), both in children and in adults [11,12]. In the obstetric population, reports of pulmonary edema secondary to tocolytic use and underlying cardiac disease can be found [1]. To our knowledge, this case represents the first report of an APEd case with bilateral hemorrhagic parenchymal infiltrations after an inadvertently supratherapeutic bolus of phenylephrine.

Adverse drug events refer to any injury related to the use of a drug, caused by a medical error or not. On the other hand, medication errors are any errors in the medication process, whether they result in adverse consequences or not. Their incidence in anesthesia practice is not well-established, but according to a limited number of prospective studies, it ranges from 0.33% to 0.73% per case [13]. The largest categories of medication error seem to be: wrong medication, overdose, and incorrect administration route. Frequent causes of overdose involve misunderstanding or preconception of the dose and dilution errors [13]. Besides the morbidity and mortality associated, adverse drug events significantly add to the financial burden of healthcare systems and families. For healthcare professionals, medico-legal consequences are a frightening reality, as doctors are being increasingly held accountable for those events. Fortunately, in this case, the patient evolved favorably within a few days and there were no harmful consequences to the neonate.

Since these errors are preventable and could have fatal consequences, patient safety should be a priority in the operating room. The case demonstrates how a quick review of events is crucial for errors to be identified, which can prevent future accidents, as well as foster team building and trust among workers. We would like to stress that notification of this adverse drug event to the pharmaceutical company should have been carried out afterward to bring attention to a formula with a high potential for error. A debriefing was the ATMs' priority, however, which allowed clarification of residual doubts, implementation of new strategies, and improvement in communication and teamwork.

## Conclusions

This case illustrates an adverse drug event, highlighting the high morbidity associated with these accidents and underlining the importance of their prevention within a complex healthcare environment. The case also highlights that careful management of hemodynamics is imperative in full-term pregnancies.
